# Phylogenetic and genotypic characteristics of foot and mouth disease virus in cattle in Tra Vinh province, Vietnam

**DOI:** 10.14202/vetworld.2024.2858-2864

**Published:** 2024-12-19

**Authors:** Tran Duy Khang, Nguyen Phuc Khanh

**Affiliations:** Faculty of Veterinary Medicine, College of Agriculture, Can Tho University, Can Tho, Vietnam

**Keywords:** cattle, foot-and-mouth disease virus, polymerase chain reaction, Tra Vinh, Vietnam, *VP1*

## Abstract

**Background and Aim:**

Foot-and-mouth disease (FMD) is a dangerous infectious disease in livestock that rapidly spreads and causes economic losses for cattle farmers. However, the genetic characteristics of FMD virus (FMDV) strains that cause FMD in many provinces of the Mekong Delta, especially Tra Vinh province, remain unclear. Therefore, this study aimed to investigate the genotype of FMDV circulating in the Tra Vinh Province, Vietnam.

**Materials and Methods:**

Forty-five probang samples from sick cows with clinical symptoms of FMD were collected and tested using reverse transcription-polymerase chain reaction (RT-PCR) to amplify the UTR (untranslated region) segment to determine FMDV. For the gene encoding *VP1*, four FMDV-positive samples with precise electrophoresis results were amplified and sequenced using the RT-PCR technique. A phylogenetic tree was established to analyze the relationship between the detected FMDV and GenBank sequences. Nucleotide and amino acid sequences were analyzed to identify mutation sites in the *VP1* gene of the detected strains and GenBank sequences.

**Results:**

Phylogenetic analysis showed that all four detected strains belonged to serotype O, topotype SEA/Mya-98. The results of *VP1* gene analysis showed that the strains detected in Tra Vinh province belonged to serotype O and had a high nucleotide similarity rate with strain O/MYA/7/98 (93.83%–96.22%). These strains shared high homology with strains from Laos and Thailand but low homology with vaccine strain O/Manisa (DI431238.1). In addition, changes of 27 amino acids were discovered in the VP1 protein of the FMDV strains, several of which were significant FMDV neutralization-related antigenic determinants. These results imply that existing vaccination may not protect against the FMDV strains circulating in the Tra Vinh Province, Vietnam.

**Conclusion:**

This study showed that these strains belong to serotype O, topotype SEA/Mya-98. In addition, mutations at 27 amino acid positions on the *VP1* gene of these strains reduce the effectiveness of disease prevention with currently used vaccines.

## Introduction

Foot-and-mouth disease (FMD) is a severe and highly contagious disease that affects cloven-hoofed animals, including cattle, sheep, goats, camelids, deer, and pigs [1]. The epidemiology of FMD is complex due to the virus’s rapid spread across a wide range of hosts, various serotypes, and subtypes, and its widespread spread across three continents: Africa, Asia, and Latin America [2]. The disease is caused by the FMD virus (FMDV), which belongs to the family *Picornaviridae* and genus *Aphthovirus*. FMDV has a polyhedral structure with a diameter of 25–30 nm. The viral RNA structure is surrounded by a capsid comprising 60 morphological units (capsomers). Each morphological unit comprises four structural proteins: VP1, VP2, VP3, and VP4. The structural proteins VP1, VP2, and VP3 are located on the surface of the capsid, whereas the structural protein VP4 is inside the capsid. There are seven different serotypes of FMDV, each with a diversity of topotypes, genetic lineages, and strains based on the degree of sequence differences of the *VP1* gene [3, 4].

Nucleotide sequences of the *VP1* coding region have been used to determine the strain and genetic characteristics of FMDV because the structural protein VP1 plays a key role in determining the attachment and entry of the FMDV, affecting the protective immunity and serotype specificity of the FMDV [5, 6]. Phylogenetic analysis based on *VP1* sequences is widely used to analyze evolutionary dynamics, epidemiological relationships between strains or subtypes, and traceability and outbreak risk prediction [7, 8]. Research on FMDV in Vietnam showed that there was a circulation of FMDV strains, including O/ME-SA/Pan Asia, O/SEA/Mya98, O/Cathay, O/ME-SA/Ind2001d, and A/Sea/97 [9]. However, the studies conducted so far have only focused on specific regions of the country. FMD still occurs in cow-raising households in the Mekong Delta, where studies on FMDV are still limited, especially in localities with developed beef farming, such as Tra Vinh province.

Therefore, this study aimed to investigate the genotype of FMDV circulating in the Tra Vinh Province, Vietnam.

## Materials and Methods

### Ethical approval

The study complied with the institutional rules for the care and use of laboratory animals and was approved by the Ministry of Agriculture and Rural Development of Vietnam (TCVN 8402:2010).

### Study period and location

The study was conducted from June 2023 to June 2024 in Tra Vinh province, Vietnam. The samples were analyzed at the Veterinary Epidemiology Laboratory of Can Tho University, Vietnam.

### Samples collection

Probang samples (n = 45) were obtained from cattle with clinical symptoms of FMD from livestock households in the Tra Vinh province of the Mekong Delta, Vietnam. After collection, the samples were dissolved in an Eppendorf tube containing 0.5 mL of phosphate-buffered saline (pH = 7.4) and stored at −80°C until analysis.

### Reverse transcription-polymerase chain reaction (RT-PCR)

RT-PCR was used to detect FMDV according to the World Organization for Animal Health guidelines. According to the manufacturer’s instructions, RNA was extracted from probang samples using an RNA extraction kit (NEXprepTM, Korea). The extracted RNA was used to synthesize cDNA using the Sensi FAST™ cDNA Synthesis kit (Bioline, UK). Briefly, the reaction mixture contained 5 μL extracted RNA template, 1 μL of reverse transcriptase, 4 μL of 5× TransAmp Buffer, and 10 μL of ultra-pure water. The reaction was incubated in a thermocycler (Bio-Rad, USA) programmed at 25°C for 10 min, 42°C for 15 min, and 85°C for 5 min. The cDNA products were immediately used for PCR, which was performed using the MyTaq™ DNA Polymerase kit (Bioline, UK) according to the manufacturer’s instructions. In brief, the PCR amplification reaction was carried out in 0.2 mL tubes. 50 μL reaction contained 25 μL of MyTaq Mix 2X, 1 μL of each primer (forward primer GCCTG-GTCTT-TCCAG-GTCT, reverse primer CCAGT-CCCCT-TCTCA-GATC [10]), 17 μL nuclease-free water, and 6 μL of the cDNA templates.

### Sequencing and phylogenetic analysis

To determine the FMDV genotype detected at the survey location, a pair of primers that amplify the *VP1* gene was designed, including forward primer: GACTT(T/C)GAG(C/T)T(A/G)CG(C/G/T)(T/C)T(A/G)CC and reverse primer: GGGTTGGACTC (A/C/G)ACGTC(T/C)CC. The thermal cycle used for the PCR reaction included the following stages: Pre-denaturation at 95°C for 3 min; 35 cycles of denaturation at 95°C for 15 s, annealing at 60°C for 15 s, extension at 72°C for 30 s; and ended elongation at 72°C for 3 min. The amplified RT-PCR products were visualized using 1.5% agarose gel electrophoresis. Positive amplicons were purified using the ISOLATE II PCR and Gel kit (Bioline) according to the manufacturer’s instructions. After purification, the samples were sent to a commercial laboratory for Sanger sequencing.

### Phylogenetic analysis

The complete *VP1* genetic sequences of the four FMDV strains were analyzed to identify the topotype. A phylogenetic tree was constructed based on the nucleotide sequences using MEGA version 10.2.6 (https://www.megasoftware.net) using the maximum likelihood method of phylogenetic tree construction with 1000 bootstrap replications. The nucleotide and amino acid sequences of the partial *VP1* of 4 detected FMDVs were aligned and compared with those of other reference and vaccine strains using the ClustalW multiple alignment method in BioEdit 7.2.0 (http://www.mbio.ncsu.edu/BioEdit/bioedit.html). Mutations and insertions were determined according to the results of pairwise comparisons between detected FMDVs and reference FMDV strains consisting of O/Manisa (DI431238.1), O/MYA/7/98 (DQ164925.1), O/VN/QB88/2009 (GU582115.1), O/VN/QN132/2009 (GU582120.1), O/LAO/3/2008 (HQ116181.1), O/TAI/1/2009 (HQ116256.1), O/VIT/7/2006 (HQ116290), MYA/3/2010 (JQ070319.1), VIT/2/2010 (JQ070322.1), O/VIT/25/2010 (KT153126.1), and O/VN1/2014 (MH845413).

## Results

### Characterization of *VP1* sequence

The results of analyzing 45 probang samples by RT-PCR using primer pairs to amplify the UTR segment showed that four samples were positive for FMDV, accounting for 8.89%. In addition, using FMDV-specific primers, all four samples (O/VN/CTU/TV01, O/VN/CTU/TV02, O/VN/CTU/TV03, and O/VN/CTU/TV04) were found to be positive for FMDV by RT-PCR. The nucleotide sequences of the *VP1* coding region of O/VN/CTU/TV01, O/VN/CTU/TV02, O/VN/CTU/TV03, and O/VN/CTU/TV04 have been submitted to National Center for Biotechnology Information (NCBI) (Accession No. PP897840.1, PP897841.1, PP897842.1, and PP897843.1, respectively).

Results of sequence analysis showed that the length of the *VP1* sequence of the FMDV strains O/VN/CTU/TV01, O/VN/CTU/TV02, O/VN/CTU/TV03, and O/VN/CTU/TV04 was 633 bp. In addition, substitutions at 77 positions were detected in the nucleotide sequences of the four strains (Table-1). No deletion, insertion, or inversion mutations were detected in these strains.

**Table-1:** Nucleotide position differences between four detected foot-and-mouth disease virus strains.

No.	Nu	(1)	(2)	(3)	(4)	No.	Nu	(1)	(2)	(3)	(4)	No.	Nu	(1)	(2)	(3)	(4)
1	15	C	T	T	C	27	234	G	G	A	A	53	453	G	G	A	A
2	30	T	C	C	T	28	258	C	T	C	C	54	458	A	C	A	A
3	45	T	T	C	C	29	270	G	G	G	A	55	465	G	G	A	G
4	51	C	C	T	C	30	285	G	A	G	G	56	472	C	C	T	C
5	57	T	T	C	C	31	286	G	G	A	A	57	477	G	G	A	G
6	60	T	T	C	C	32	291	C	T	T	T	58	480	T	T	C	C
7	81	C	T	C	C	33	292	T	T	C	C	59	483	T	T	C	T
8	84	T	C	T	T	34	295	A	A	G	G	60	486	T	T	C	C
9	90	A	A	G	C	35	306	C	C	C	T	61	513	T	C	T	T
10	99	A	G	A	G	36	318	G	G	C	G	62	514	C	C	A	C
11	106	T	T	C	C	37	324	C	T	C	C	63	531	G	C	G	G
12	125	G	T	T	T	38	333	G	G	G	T	64	555	G	A	A	A
13	126	T	C	T	T	39	336	C	C	T	T	65	564	T	T	C	T
14	140	A	C	A	A	40	349	T	C	C	C	66	567	C	G	A	G
15	144	T	T	T	C	41	354	T	C	T	C	67	570	T	C	T	T
16	150	A	G	G	G	42	366	A	G	A	A	68	574	C	T	T	C
17	168	T	C	T	C	43	384	T	C	C	C	69	585	T	T	T	C
18	172	C	C	T	T	44	396	G	G	A	A	70	593	G	G	A	A
19	180	C	T	T	T	45	398	A	G	A	A	71	596	C	A	C	C
20	181	C	C	T	T	46	405	A	A	G	G	72	600	G	A	G	A
21	186	A	G	G	G	47	424	A	A	A	G	73	606	C	A	A	A
22	201	C	T	T	T	48	429	C	A	C	C	74	610	G	A	A	G
23	216	T	T	C	C	49	430	A	G	G	G	75	613	T	A	A	A
24	222	T	C	T	C	50	438	T	C	T	T	76	621	A	G	A	A
25	228	A	A	A	G	51	447	A	G	G	G	77	627	G	A	G	G
26	231	A	A	G	G	52	451	T	C	C	T						

Nu=Nucleotide; (1)=O/VN/CTU/TV01; (2)=O/VN/CTU/TV02; (3)=O/VN/CTU/TV03; (4)=O/VN/CTU/TV04

### Phylogenetic and pairwise sequence comparison analysis of *VP1* sequence

**Figure-1:**
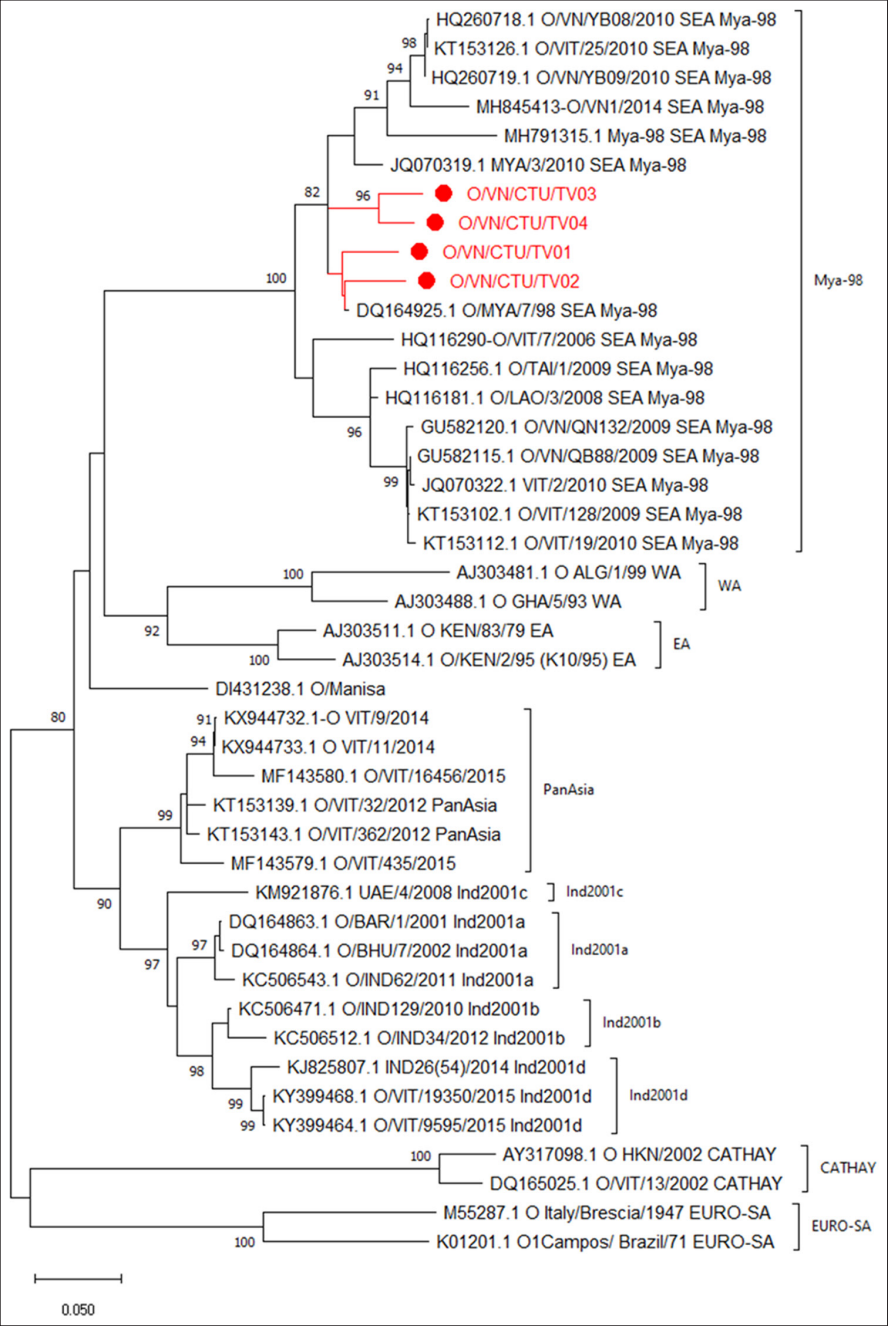
Phylogenetic trees of foot-and-mouth disease virus based on the nucleotide sequences of *VP1* gene.

A total of 39 *VP1* sequences (15 sequences of SEA topotype, lineage Mya-98 and 24 sequences of other topotypes in serotype O) were retrieved from the NCBI. The 43 *VP1* sequences were used to construct the phylogenetic tree with a bootstrap value of 1000 replicates. The genetic relationships of the detected FMDV strains with the other FMDV strains are shown in the phylogenetic tree (Figure-1). Based on the full-length *VP1* gene, a phylogenetic tree demonstrating that all four detected strains O/VN/CTU/TV01, O/VN/CTU/TV02, O/VN/CTU/TV03, and O/VN/CTU/TV04 were clustered into the same group of O serotype, SEA topotype, and Mya-98 lineage, as indicated in Figure-1.

The nucleotide sequence of the complete *VP1* gene was 633 bp in all virus strains. The comparison of the nucleotide sequences of four studied strains revealed 77 mutations in 633 positions, accounting for 12.16%. The FMDV strains in this study shared high similarity (91.06%–95.54%) (Table-2). Results of comparing the similarity between 4 FMDV strains O/VN/CTU/TV01, O/VN/CTU/TV02, O/VN/CTU/TV03, and O/VN/CTU/TV04 detected in the Tra Vinh Province with two reference strains including O/MYA/7/98 (DQ164925.1) and MYA/3/2010 (JQ070319.1) showed that these strains have high similarity with each other with a similarity ratio of 91.98%–96.2%. In addition, the rate of nucleotide similarity between the strains O/VN/CTU/TV01, O/VN/CTU/TV02, O/VN/CTU/TV03, and O/VN/CTU/TV04 with reference strain O/MYA/7/98 (DQ164925.1), which was first detected in Myanmar in 1998, was very high, with a percentage of 93.83%–96.22%.

**Table-2:** Nucleotide and amino acid sequence identities (%) between the strains used in this study and those of the reference sequences.

Accession number	Nucleotide				Amino acid			
	
	1	2	3	4	1	2	3	4
Within field strains, the following expressions are considered								
PP897840.1	91.06–95.54				94.62–98.56			
PP897841.1								
PP897842.1								
PP897843.1								
Field strains for commercial vaccines in the Tra Vinh province (Vietnam)								
DI431238.1	80.99	79.70	81.79	80.89	89.46	89.46	92.08	92.08
Field strains to other reference strains of the same topotype								
DQ164925.1	95.88	96.22	93.83	93.83	97.10	97.10	98.08	97.59
GU582115.1	88.61	89.13	88.45	88.29	95.12	95.12	96.12	95.62
GU582120.1	88.23	88.37	88.07	87.90	94.62	94.62	95.62	95.12
HQ116181.1	89.76	90.10	88.87	88.89	94.62	94.62	96.12	95.62
HQ116256.1	88.81	89.15	87.91	87.93	92.59	92.59	94.62	94.12
HQ116290	91.16	89.47	88.15	88.54	93.10	93.10	94.12	93.61
JQ070319.1	92.70	92.31	91.98	93.07	94.12	94.12	97.10	96.61
JQ070322.1	88.24	88.76	88.45	88.29	95.12	95.12	96.12	95.62
KT153126.1	92.54	92.89	89.79	90.36	95.12	95.62	96.61	96.12
MH845413	90.27	91.40	87.79	88.95	92.08	93.61	93.61	93.61

1=O/VN/CTU/TV01; 2=O/VN/CTU/TV02; 3=O/VN/CTU/TV03; 4=O/VN/CTU/TV04

### Variations in the amino acids of the *VP1* gene

**Figure-2:**
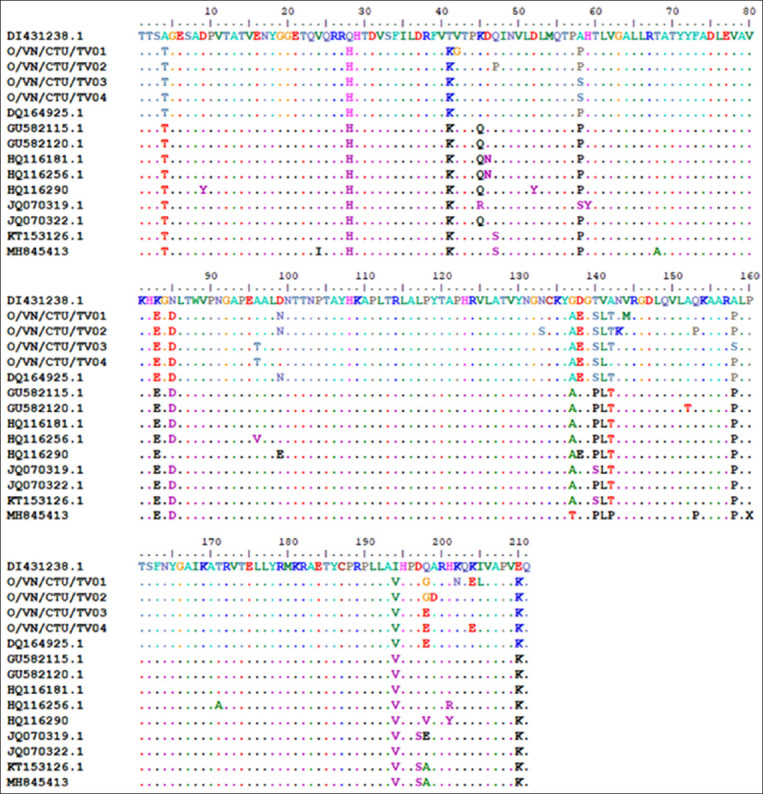
Amino acid sequences of antigenic regions in *VP1* of foot-and-mouth disease virus. Amino acid sequence alignments of *VP1* in four detected strains and a reference vaccine strain. Dots indicate amino acid positions; letters indicate amino acid changes.

Based on our analysis of the *VP1* amino acid sequences, 16 substitutions were found among the FMDV strains in this study. Compared with the amino acid sequences of the O1/Manisa virus strain, which is commonly used in Vietnam’s FMD vaccines, we found 27 amino acids substituted between the detected strains and the FMDV vaccine strains (Figure-2). Moreover, several substitutions were identified in critical antigenic determinants involved in the neutralization of FMDV, including amino acids 137, 138, 140–144, 153, and 158 at amino acids 136–160.

## Discussion

The presence of FMD in Vietnam is a significant barrier to the sustainable development of the livestock industry due to its harmful effects on productivity and the trade of animals and livestock products. A national vaccination program and uniform vaccine strain policy have been established in Vietnam. Under this program, cattle, buffaloes, and pigs are protected against FMD by receiving a biannual injection of an inactivated FMD vaccine. In developing nations, especially Vietnam, where FMD is endemic, the disease’s complicated epidemiology presents a significant obstacle to its control. According to Gunasekara *et al*. [9], FMDV serotype O is the leading cause of FMD in Vietnam. We studied molecular genetics of FMDV serotype O using the maximum likelihood method. The phylogenetic tree serves as a foundation for molecular epidemiological research and the determination of the serotypes of FMDV strains by visually representing their parental relationships [11, 12]. *VP1*, a highly variable region of the FMDV genome, is widely employed as the primary gene of choice for phylogenetic analysis and serotype determination worldwide [5, 6].

Based on the full-length *VP1* gene, a phylogenetic tree demonstrated that all four FMDV strains obtained in Tra Vinh province, Vietnam, were clustered into a branch of the O serotype, SEA topotype, and lineage Mya-98. O/SEA/Mya-98 is a specific strain of Southeast Asia isolated from many countries, especially in Vietnam [13, 14]. SEA was the most frequently detected topotype with two lineages, Mya-98 and Cam-94 [15].

Another point worth noting is that FMDV strains O/VN/CTU/TV01, O/VN/CTU/TV02, O/VN/CTU/TV03, and O/VN/CTU/TV04 have very high genetic similarity rates (93.81%–96.19%) with the reference strain O/MYA/7/98 (DQ164925.1). The presence of Mya-98 FMDV strains has caused significant economic losses in many Asian countries, such as Vietnam (2006), Japan, South Korea (2010), and North Korea (2011) [16]. Regarding genetics, the detected strain is closely related to some Lao and Thai species (HQ116181.1 and HQ116256.1, respectively), which appear to share a common ancestor. These results suggest that FMDV is spread through cattle trade between these countries. Bertram *et al*. [17] have demonstrated that cattle trade is a mode of transmission of FMDV.

Another factor to consider is the discovery of amino acid mutations in the four studied strains. Among the FMDV strains examined in this study, 16 amino acid substitution mutations were present in the *VP1* gene. When comparing the amino acid of the *VP1* gene sequence between four field virus strains and the O1/Manisa virus strain commonly used in FMD vaccines in Tra Vinh province (Vietnam), 27 mutated amino acid substitutions were detected. Several substitutions were identified in critical antigenic determinants involved in the neutralization of FMDV, including amino acids 137, 138, 140–144, 153, and 158 at amino acids 136–160. Amino acids 144, 148, and 154 play critical roles in the neutralizing capabilities of this FMDV antigenic site located in amino acids 140–160 of *VP1* [18]. The *VP1* gene encoding the VP1 protein contains at least two critical immunogenic sites at amino acid positions 141–160 and 200–213. Amino acid positions 141–160 contain arginine-glycine-aspartic acid, which is responsible for viral attachment to host cells via the integrin receptor [19]. Moreover, T-cell epitopes in *VP1* were found in several studies at amino acids 4–13, 106–115 [20], and 21–40 [21]. In regions of T-cell epitopes, amino acid changes were found in this study, including amino acids 4 at amino acids 4–13 and amino acids 28 at amino acids 21–40. In brief, multiple changes in crucial amino acid positions of the FMDV *VP1* gene were identified by sequence analysis, which may explain why current vaccines are not as effective against detected strains of the natural FMDV strains [22]. Mutations in the VP1 protein can prevent FMDV vaccinations from providing cross-serotype protection [23, 24].

In the southern provinces of Vietnam, especially the Tra Vinh province, cattle farming plays an essential economic role in many livestock households. However, FMD still frequently occurs in cattle, one of many factors that reduce farmers’ economic efficiency. The results of this study once again emphasize the importance of regular surveillance of the circulation of FMDV strains and the selection of preventive vaccines appropriate for the detected strains.

### Limitations and strength of the study

The results of the *VP1* gene mutation in this study did not determine whether it affected the effectiveness of the currently used vaccine. Further studies are required to overcome this limitation and demonstrate the effectiveness of vaccines. However, this study demonstrates notable strengths through its investigation of the genetic characteristics of FMDV in Tra Vinh province, a region with a substantial livestock industry but limited FMD research. By focusing on *VP1* gene variations, the study provides valuable insights into the virus’s evolution, thereby contributing to the development of more effective disease control strategies.

## Conclusion

In Vietnam, FMD still occurs frequently, even though the FMD vaccination strategy is maintained annually. The analysis of the nucleotide and amino acid sequence characteristics of the *VP1* gene showed that the detected strains were clustered into a branch of serotype O (SEA/Mya-98). The presence of many amino acid mutations in the *VP1* gene among FMDV strains indicates that these mutations may have contributed to the recent outbreaks of FMD in Tra Vinh province, Vietnam. The findings can contribute to a better understanding of the genetic diversity of FMDV in the studied area and offer additional information for the veterinary agencies in Tra Vinh province.

## Authors’ Contributions

NPK: Conceptualized and designed the study. TDK: Collected field samples. TDK and NPK: Performed molecular experiments and analyzed and interpreted the data. TDK: Wrote the first draft. TDK and NPK: Revised the manuscript. Both authors have read and approved the final manuscript.
